# Improved conservation of callus and rhizome microcuttings of *Podophyllum hexandrum* germplasm using the slow growth storage approach

**DOI:** 10.1038/s41598-025-13729-2

**Published:** 2025-08-01

**Authors:** Zahoor Khan, Bushra Khan, Syed Tanveer Shah, Javaid Iqbal, Abdul Basit, Monsif Ur Rehman, Mian Afaq Ahmad, Muhammad Fahim, Muhammad Farhan Saeed, Árpád Székely, Aftab Jamal

**Affiliations:** 1 Department of Environmental Sciences, University of Peshawar, Peshawar, KP 25130 Pakistan; 2 Department of Agriculture, Faculty of Biological and Health Sciences, Hazara University, Mansehra, KP 21120 Pakistan; 3 Department of Environmental Sciences, University of Lakki Marwat, Lakki Marwat, KP 28420 Pakistan; 4 Floricultural Biotechnology Lab, Department of Horticultural Science, Kyungpook National University, Daegu, 41566 South Korea; 5 Institute of Biotechnology and Genetic Engineering, The University of Agriculture-Peshawar, Peshawar, Khyber Pakhtunkhwa 25130 Pakistan; 6 Centre for Omic Sciences, Islamia College University Peshawar, Peshawar, KP 25130 Pakistan; 7 Department of Environmental Sciences, COMSATS University Islamabad, Vehari-Campus, Vehari, 61100 Pakistan; 8 Research Centre for Irrigation and Water Management, Institute of Environmental Sciences, Hungarian University of Agriculture and Life Sciences, Anna-Liget str. 35, Szarvas, 5540 Hungary; 9 Department of Soil and Environmental Sciences, Faculty of Crop Production Sciences, The University of Agriculture, Peshawar, 25130 Pakistan; 10 Key Laboratory of Arable Land Conservation (Middle and Lower Reaches of the Yangtze River), Ministry of Agriculture, College of Resources and Environment, Huazhong Agricultural University, Wuhan, 430070 China

**Keywords:** Osmotic regulators, Spermidine, Rhizome microcuttings, In vitro conservation, Slow growth storage (SGS), Calcium pantothenate, Tissue viability, Biotechnology, Plant sciences

## Abstract

*Podophyllum hexandrum* Royle (syn. *Podophyllum emodi* Wall.), commonly known as Himalayan mayapple, is an endangered medicinal plant recognized as the primary natural source of podophyllotoxin, a potent compound with anticancer and antiviral properties. In this study, we developed an optimized protocol for the long-term preservation of *P. hexandrum* germplasm using a slow growth storage (SGS) technique, successfully preserving the viability and genetic stability of both callus and rhizome cuttings. In vitro cultured callus and rhizome microcuttings of *P. hexandrum* were conserved using the slow growth storage (SGS) technique in Murashige and Skoog (MS) medium under cold conditions (5 °C), supplemented with different concentrations of sucrose, mannitol, and sorbitol in combination with calcium pantothenate and spermidine, to induce slow growth and maintain tissue viability. It was observed that sorbitol (5.5%) combined with spermidine (2 mg L^−1^), calcium pantothenate (3 mg L^−1^), and 6-benzylaminopurine (BA) (1.5 mg L^−1^) showed better efficacy than the mannitol (6.5%) combination in preserving and regenerating callus and rhizome microcuttings. In contrast, the combination with sucrose (6.5%) was the least effective. This study developed an effective in vitro protocol for conserving *P. hexandrum*, an endangered medicinal plant, through slow growth storage. A medium containing sorbitol, mannitol, spermidine (2 mg L^−1^), and calcium pantothenate (2 mg L^−1^) enhanced tissue viability, stress tolerance, and long-term survival of callus and rhizome explants while maintaining genetic stability during cold storage. These findings suggest that this protocol provides a reliable approach for the ex-situ conservation of *P. hexandrum*, ensuring the availability of genetically stable plant material for future research and medicinal use. This is the first report on the germplasm conservation of callus and rhizome microcuttings of *P. hexandrum* grown in Pakistan using the slow growth technique.

## Introduction

*Podophyllum hexandrum Royle* is an essential source of podophyllotoxin, which plays a crucial role as a precursor in the chemical synthesis of the anti-cancer drugs etoposide and teniposide^1^. Several previous studies have reported the medicinal properties of *P. hexandrum*, which is used to treat hepatic disorders, ulcers, tuberculosis, constipation, mental disorders, and wounds^2^. The rhizome extract of *P. hexandrum* is known to inhibit the growth of *Candida albicans* and *Aspergillus niger*^1,3^. Podophyllotoxin, a potent cytotoxic compound found in *P. hexandrum*, is not approved by the U.S. FDA for systemic use due to its toxicity^4^,. However, several derivatives of podophyllotoxin have received FDA approval for prescription-based medical use. Notably, podofilox (0.5% topical gel), marketed as Condylox^®^, is approved for treating external genital warts caused by HPV. Semi-synthetic derivatives like etoposide (VePesid^®^) and teniposide (Vumon^®^) are approved for chemotherapy, including testicular cancer, small-cell lung cancer, and acute lymphoblastic leukemia^2,5–8^. However, crude extracts of *P. hexandrum* or direct use of podophyllotoxin have not been granted GRAS (Generally Recognized as Safe) status by regulatory authorities like the FDA and EFSA. *P. hexandrum* and its crude extracts have no established concentration or safe intake levels due to their toxicity. Its derivatives are approved only as prescription drugs. Regulatory bodies like the FDA, FAO/WHO, and EFSA have not approved *P. hexandrum* for dietary use, and it lacks GRAS status^4,9,10^. Additionally, *P. hexandrum* functions as a natural insecticide with eco-friendly potential; its dichloromethane extract, which is rich in podophyllotoxin, effectively targets *Drosophila melanogaster* larvae^11^. The symbiotic relationship between plant and mycorrhizal fungi is essential for survival of fungi, hence highlighting the plant’s role in preserving ecosystem stability and biodiversity. Additionally, arbuscular mycorrhizal association significantly influences plant nutrition, soil chemistry^12^. *P. hexandrum* has been recognized for its sensitivity to environmental stress and disturbances, particularly heavy metal contamination, which supports its use as a bioindicator in alpine and subalpine ecosystems. It hyperaccumulates metals like Pb, Cd, and Zn in its tissues, reflecting soil pollution levels. Under metal stress, it shows elevated oxidative markers ROS, lipid peroxidation and increased antioxidant enzymes (SOD, CAT, POD). Notable changes include reduced root and shoot biomass, leaf chlorosis, altered chlorophyll content, and decreased photosynthetic efficiency, which have been observed in both controlled and field settings^12^. Despite its high medicinal and socio-economic value, *P. hexandrum* is under serious threat due to overexploitation, unregulated collection, and habitat degradation^13^. The rising global demand for podophyllotoxin, coupled with the plant’s prolonged juvenile phase, poor fruit set, and low seed germination, has severely reduced wild populations. Natural regeneration is further constrained by harsh winters, a short growing season, and irregular flowering, all of which limit its recovery in the wild^14^. *P. hexandrum* is currently listed as Endangered on the IUCN Red List, assessed in 2024 under criteria A2bd + 3 cd + 4 cd, reflecting at least a 50% population decline over the past three generations due to overharvesting, habitat loss, and climate change (IUCN Red List, 2024)^15,16^.

In vitro techniques such as tissue culture, genetic engineering, and biotransformation are powerful tools for the rapid propagation and long-term conservation of plant germplasm. For effective germplasm preservation, these modern biotechnological approaches are often integrated with conventional methodsl^17^. For endangered medicinal plants like *P. hexandrum*, the integration of sustainable in vitro conservation strategies with ethical and responsible sourcing practices is crucial to ensure genetic stability, viability, and ecological sustainability^18^.

Among the available conservation techniques, Slow Growth Storage (SGS) and cryopreservation are two significant approaches for safeguarding *P. hexandrum*. SGS is a cost-effective and practical strategy for medium-term conservation, particularly suitable for species with recalcitrant seeds and high sensitivity to freezing, such as *P. hexandrum*^19^. It enables the maintenance of viable in vitro cultures under reduced growth conditions such as low temperature, limited light, or osmotic regulation thereby reducing the frequency of subculturing while preserving genetic integrity^20,21^. In contrast, cryopreservation is more appropriate for long-term storage, but it requires advanced infrastructure, cryogenic facilities, and highly optimized protocols, which may not be feasible in resource-limited contexts. Therefore, SGS presents a more accessible and reliable option for conserving critically endangered medicinal plants like *P. hexandrum*^22^.

The slow growth storage (SGS) technique minimizes space, labor, and costs while reducing the risks of germplasm loss and contamination during subcultures^23,24^. Operating as an in-vitro technique, SGS contributes to ex-situ conservation by reducing plant metabolism and extending subculture intervals without compromising genetic uniformity or quality^25,26^. This approach aligns with sustainable conservation practices and supports the broader strategy of preserving genetic resources for maintaining ecosystems and promoting human well-being. Manipulating cultivation factors such as temperature, light, and nutrients leads to the creation of slow growth cultures^27,28^.

Osmotic regulators, such as mannitol, sorbitol, and sucrose, induce stress that limits in vitro explant growth by reducing water availability^29–31^. These agents, commonly used in the conservation of plant genetic resources, lower growth rates and metabolic activity, thereby extending subculture intervals and minimizing the risk of genetic instability^20,23,27^. Calcium pantothenate (CaP) and spermidine, serving as growth regulators, influence the biosynthesis of organic osmolyte, safeguarding plant cells from dehydration and oxidative damage while maintaining cell viability^32,33^. The slow-growth conservation technique has been successfully applied to a range of plant species, including Date Palm, *Phoenix. dactylifera*, *Garcinia. indica*, *Moehringia jankae Griseb*,*Fraser. photinia*, *Podophyllum. peltatum*, *Dioscorea alata L*., and *Chlorophytum. borivilianum*^32,34–38^. The effectiveness of such techniques largely depends on several factors, including plant species, explant type, genotype, and physiological conditions^23,27,38–40^. The slow-growth conservation technique not only helps in germplasm conservation of *P. hexandrum* but also support sustainable biotechnological applications such as genetic transformation and podophyllotoxin production. Despite the promising role of in vitro biotechnological strategies for podophyllotoxin synthesis, the successful propagation and long-term conservation of threatened medicinal plants remain difficult, with only limited studies protocols^2,24,41–43^. Previous studies on *P. hexandrum* conservation and other endangered medicinal plants have predominantly emphasized micropropagation of shoots or seed banking, with limited focus on long-term preservation methods like slow-growth storage (SGS)^21–24,41^. Moreover, few efforts have optimized conservation protocols for critical explants such as callus and rhizome tissues, which offer greater sustainability for secondary metabolite production^2,3,8,16,17,41,44,45^. Earlier studies on the in vitro conservation of *P. hexandrum* was limited by low regeneration rates, short-term viability (≤ 6 months), and reliance on sucrose without testing osmotic agents like sorbitol or mannitol. Additives such as spermidine and calcium pantothenate were rarely used, and recovery protocols lacked development, leaving long-term regeneration potential unclear. This study addressed gaps by optimizing osmotic regulator concentrations, incorporating synergistic growth supplements, and evaluating survival and regeneration responses over extended storage durations (4–12 months). The originality of our study lies in the development of a first-of-its-kind slow growth storage (SGS) protocol specifically tailored for the conservation of *P. hexandrum* germplasm (callus and rhizome microcuttings) collected from Pakistan. Previous studies have rarely focused on in vitro long-term conservation strategies for this endangered species, particularly in the South Asian Himalayan region. Unlike earlier reports that primarily addressed propagation or pharmacological profiling, our study introduces an optimized, reproducible SGS medium that enhances tissue viability and stress tolerance during cold storage^14,24,41,42^. Specifically, the aim was to develop an efficient in vitro slow-growth conservation protocol for *P. hexandrum* by assessing the effects of osmotic agents and growth supplements on the survival and regeneration of callus and rhizome explants under cold storage conditions.This research seeks to enhance our understanding and provide innovative solutions in an area that remains unexplored in Pakistan.

## Materials and methods

### Plant material and culture initiation

**Fig. 1:**
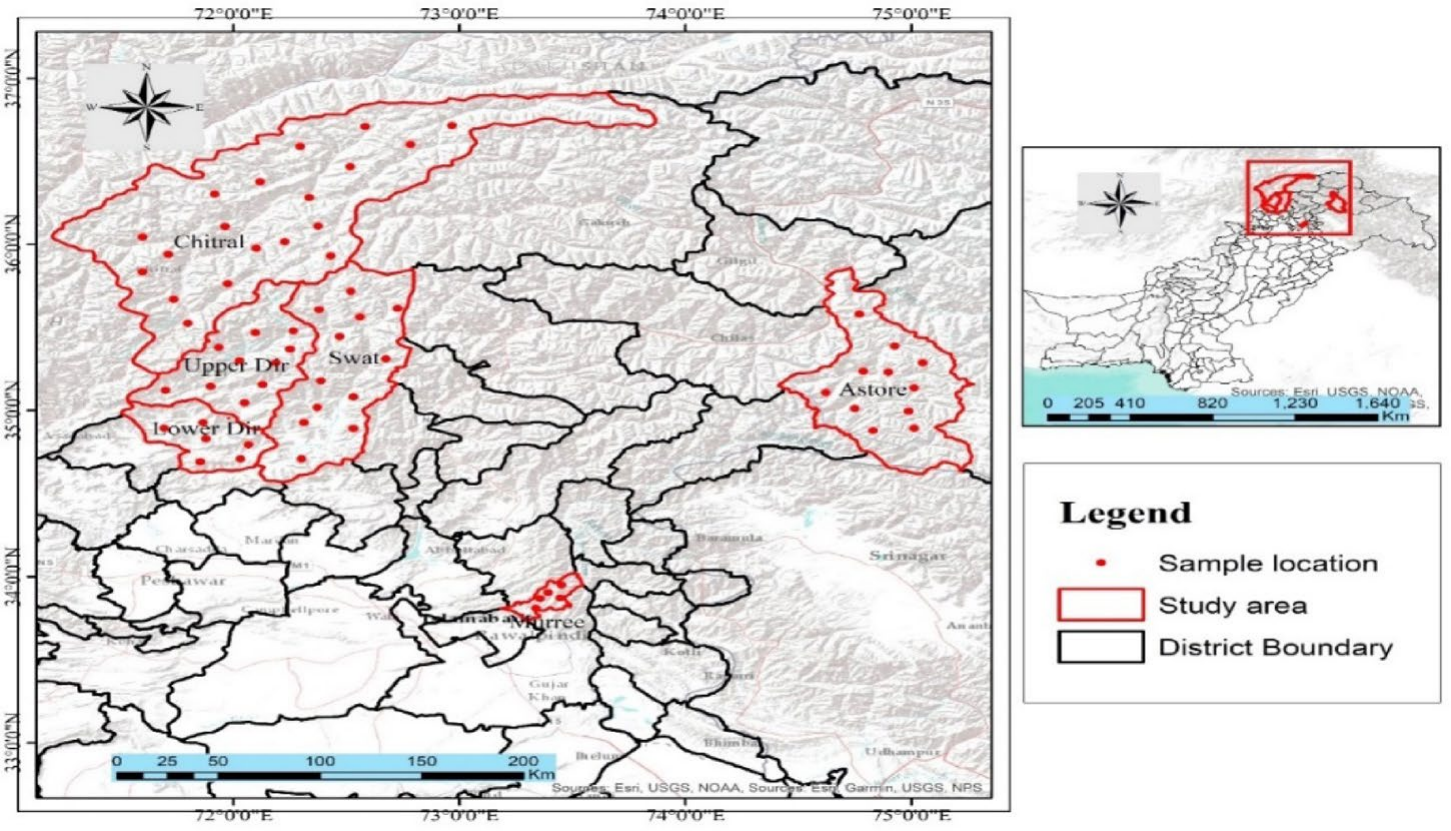
Maps illustrating the sampling sites and distribution of *P. hexandrum* in various locations across Swat, Upper Dir, Lower Dir, Chitral, Astore, and Murree District in KPK, Pakistan.

Plant materials of *P. hexandrum* were collected from their natural habitats in the Swat Gabral and Utror valleys, Astore, Hazara, Dir, Murree Hills, and Chitral district of Khyber Pakhtunkhwa province in Northern Pakistan, using a random sampling approach (Fig. 1). A pooled sample approach was adopted morphologically similar and healthy explants from different locations were combined to initiate cultures. This mixed-genotype method was chosen to reflect the general response of *P. hexandrum* populations. For tissue culture initiation, explants (rhizome segments and nodal buds) were surface-sterilized and cultured on Murashige and Skoog (MS) medium supplemented with growth regulators. The plant was identified by Dr. Ghulam Jailani, assigned a voucher number (2001), and deposited at the Herbarium, Department of Botany, University of Peshawar, Pakistan.The research experiments were conducted at the Plant Tissue Culture and Germplasm Conservation Division, Institute of Biotechnology and Genetic Engineering (IBGE), The University of Agriculture, Peshawar.

### Sterilization of plant materials

Leaf and rhizome microcuttings with active buds were obtained from the collected plant material and used as the explant source. Leaf explants (0.5 × 0.5 cm to 1 × 1 cm) and rhizome microcuttings (0.5–1.0 cm in length, 3–5 mm in diameter) were prepared under sterile conditions, thoroughly washed with tap water, and then immersed in 20% Tween-20 solution for 5 min to eliminate contaminants. The explants were subsequently immersed in a 20% sodium hypochlorite solution for 10 min, followed by surface sterilization with a 0.1% mercuric chloride solution for 5 min. Finally, the sterilized explants were rinsed three times with sterile distilled water.

### Aseptic transfer of explants

The in vitro cultures of *P. hexandrum* explants were conducted using Murashige and Skoog’s (1962) MS basal medium as the growth substrate. The MS media were supplemented with vitamins, plant growth regulators (PGRs), 3 g/l activated charcoal (AC), 3–5 g/l sucrose, and 8 g/l agar as a gelling agent. The pH of the MS media was adjusted to 5.8 using 1 N HCl and 1 N NaOH, the media were then autoclaved at 121 °C for 15 min. The leaf explants and rhizome microcuttings were transferred into conical flasks containing 250 ml and 100 ml of MS medium under aseptic conditions.

### Callus induction from the explants of *P. hexandrum*

Callus induction was initiated from *P. hexandrum* explants following the partially modified protocols established by AlMousa et al.(2022), Nadeem et al. (2000) and Zuhra et al. (2021)^31,41,42^. Explants of *P. hexandrum* were inoculated on Murashige and Skoog (MS) media supplemented with 1.5 mg L^−1^ Benzyl adenine (BA) + 0.5 mg L^−1^ 4-Dichlorophenoxy acetic acid (2,4-D) for germplasm conservation. The synergistic and antagonistic effects of these hormones on callus induction were evaluated. To prevent callus necrosis and browning, ascorbic acid (50, 100, and 150 mg L^−1^) and activated charcoal (3 mg L^−1^) were added to the MS media. Four leaf explants were cultured in each 250 mL and 500 mL conical flask containing 250 mL and 100 mL of MS medium, respectively, with one flask representing one replicate and three replicates per treatment. Conical flasks containing MS media and explants were transferred to a growth room at 25 ± 2 °C under dark conditions for 45 days. The cultures were then incubated at 25 ± 2 °C under a 16/8 h photoperiod with a light intensity of 40 µmol/m^2^/s provided by cool white, fluorescent tubes. To enhance callogenesis and avoid browning and necrosis, the calli were subcultured on fresh MS medium with the same composition and conditions every four weeks.

The following indices were recorded to evaluate the effects of treatments under slow-growth conditions. Callus survival percentage was calculated as the ratio of viable calli to total calli per treatment. Callus weight (CWT, g**)** was measured after removing surface moisture. Growth rate (GRT, mm/day) was assessed based on the linear expansion of callus tissue over time. Growth per gram (GPG, mm/g) was derived by dividing the total growth by callus weight. Callus recovery percentage (CR_Reco %) referred to the proportion of calli that resumed normal growth after storage. Callus regeneration percentage (CR Regen %) was calculated as the number of explants producing shoots or plantlets relative to the total. For rhizome explants, shoot number and shoot length (cm) were recorded after 4 weeks of subculture on regeneration media.

### In vitro germplasm conservation of callus under slow growth conditions

To evaluate the effects of different osmotic agents and growth regulators on the conservation of calli under slow-growth conditions, calli were transferred to MS-based conservation media supplemented with varying combinations of 2% sucrose, 4% mannitol, or 4% sorbitol. Each osmotic agent was applied as a separate treatment in combination with constant concentrations of spermidine (2 mg L^−1^) and calcium pantothenate (3 mg L^−1^). Half-strength MS medium was added to 0.7% agar (without growth regulators) and 3.0% sucrose (standard concentration) to be used as a control medium. The conservation medium was supplemented with osmotic agents and growth regulators in various concentrations (Table 1). The pH of the medium was adjusted to 5.8 before autoclaving. Six-week-old calli were cut into uniform pieces (~ 0.5 g each) and inoculated into 250 mL and 500 mL Erlenmeyer flasks containing 100 mL and 250 mL of conservation medium, respectively. The use of two flask sizes was based on material availability; however, the explant-to-medium ratio was proportionally maintained to ensure consistency across treatments. Three flasks per replicate were used for passaging cells and maintaining explants in culture medium. Each treatment included three replicates, with 10 callus pieces per replicate. The flasks were sealed with aluminum foil and stored in complete darkness at 5 °C for conservation periods of 4, 8, and 12 months.

**Table 1:** Composition of the conservation medium with osmotic agents and growth regulators for *P. hexandrum* callus stored in the dark at 5 °C for 4, 8, and 12 months.

Treatment code	Osmotic agent (%)	CaP (mg L^−1^)	Spd (mg L^−1^)	Temp (°C)	Time (months)
Su (2 + 0 + 0)	Sucrose 2.0	0	0	5	4, 8, 12
Su + CaP (2 + 2 + 0)	Sucrose 2.0	2.0	0	5	4, 8, 12
Su + Spd (2 + 0 + 2)	Sucrose 2.0	0	2.0	5	4, 8, 12
M (4 + 0 + 0)	Mannitol 4.0	0	0	5	4, 8, 12
M + CaP (4 + 2 + 0)	Mannitol 4.0	2.0	0	5	4, 8, 12
M + Spd (4 + 0 + 2)	Mannitol 4.0	0	2.0	5	4, 8, 12
So (4 + 0 + 0)	Sorbitol 4.0	0	0	5	4, 8, 12
So + CaP (4 + 2 + 0)	Sorbitol 4.0	2.0	0	5	4, 8, 12
So + Spd (4 + 0 + 2)	Sorbitol 4.0	0	2.0	5	4, 8, 12

Su = Sucrose; M = Mannitol; Sor = Sorbitol; CaP = Calcium pantothenate; Spd = Spermidine.

All treatments were conducted at 5 °C for specified time periods (6 or 12 months).

### Survival and viability assessment

The survival and viability of the calli were assessed at the end of each conservation period (4, 8, and 12 months). The survival percentage was calculated as the ratio of living calli to the total number of inoculated calli. Callus viability was determined using the tetrazolium test, which measures dehydrogenase activity in living cells. The calli were immersed in a 0.1% solution of 2,3,5-triphenyltetrazolium chloride (TTC) for 4 h at 37 ˚C in the dark, then rinsed with distilled water and observed under a stereomicroscope. The viable calli showed red coloration, while the non-viable calli remained colorless. The viability percentage was calculated as the ratio of viable calli to the total number of calli tested, multiplied by 100.

### Recovery and regeneration of calli

After each conservation period, the calli from each treatment were transferred to recovery medium (RM) consisting of MS with 3% sucrose, 0.8% agar, 0.5 mg L^−1^ 2,4 − D, and 1.5 mg L^−1^ BA. The cultures were incubated at 25 ± 2 ˚C under 16/8 h photoperiod with a light intensity of 50 µmol/m^2^/s. The recovery percentage was calculated as the ratio of the number of calli that resumed growth to the total number of calli transferred. The regeneration percentage was calculated as the ratio of the number of calli that produced shoots and/or roots to the total number of calli transferred. The regeneration period lasted for 4 weeks.

### In vitro germplasm conservation of rhizome explants of *P. hexandrum*

The effects of various osmotic regulator concentrations on the survival and growth of *P. hexandrum* rhizome explants (0.5–1.0 cm in length, 3–5 mm in diameter) were evaluated under minimal slow growth storage conditions (5 °C in the dark). One rhizome microcutting per flask was used to prevent cross-contamination and allow individual monitoring. Initially, sterilized rhizome microcuttings were cultured on MS medium supplemented with activated charcoal (3 mg L^−1^) and 6-benzylaminopurine (BA) (2.5 mg L^−1^) at 25 ± 2 °C under a 16/8 h light/dark photoperiod (40 µmol m^−2^ s^−1^). Subculturing was performed every 4 weeks on fresh MS medium containing 3% sucrose and 0.8% agar. After 12 weeks, the rhizome explants (1.5 to 2.3 cm in length) were transferred to conservation media composed of half-strength MS, 0.7% agar, 3% sucrose (control) or different concentrations (2.5–10.5%) of sucrose, mannitol, or sorbitol, supplemented with BA (1.5 mg L^−1^), activated charcoal (3 mg L^−1^), calcium pantothenate (CaP, 1.5 mg L^−1^), and spermidine (Spd, 1.5 mg L^−1^). Sorbitol, mannitol, and sucrose were applied as separate treatments to the rhizome microcuttings each in combination with a constant concentration of spermidine (2 mg L^−1^) and calcium pantothenate (3 mg L^−1^). All cultures were maintained at 5 °C in complete darkness for 6 and 12 months. A total of 30 distinct treatments were applied 10 for each osmotic regulator (Table 2). Each treatment consisted of three replicates, with three explants per flask. The percentage survival was assessed at 6 and 12 months based on the number of viable explants.

**Table 2:** Composition of conservation media supplemented with varying concentrations of osmotic agents for rhizome microcuttings of *P. hexandrum* stored at 5 °C in the dark.

Treatment	Osmotic agent	Con (%)	Treatment	Osmotic agent	Con (%)	Treatment	Osmotic agent	Con (%)
T1 (control)								
T2	Sucrose	3.0	T2	Mannitol	3.0	T2	Sorbitol	3.0
T3	Sucrose	2.5	T3	Mannitol	2.5	T3	Sorbitol	2.5
T4	Sucrose	3.5	T4	Mannitol	3.5	T4	Sorbitol	3.5
T5	Sucrose	4.5	T5	Mannitol	4.5	T5	Sorbitol	4.5
T6	Sucrose	5.5	T6	Mannitol	5.5	T6	Sorbitol	5.5
T7	Sucrose	6.5	T7	Mannitol	6.5	T7	Sorbitol	6.5
T8	Sucrose	7.5	T8	Mannitol	7.5	T8	Sorbitol	7.5
T9	Sucrose	8.5	T9	Mannitol	8.5	T9	Sorbitol	8.5
T10	Sucrose	9.5	T10	Mannitol	9.5	T10	Sorbitol	9.5
T11	Sucrose	10.5	T11	Mannitol	10.5	T11	Sorbitol	10.5

All treatments were uniformly supplemented with BA (1.5 mg L^−1^), activated charcoal (3 mg L^−1^), calcium pantothenate (CaP, 1.5 mg L^−1^), and spermidine (Spd, 1.5 mg L^−1^). *“Con (%) = Concentration in percent*.

Control treatment represents only MS media except plant growth regulators and osmotic agents.

### Regeneration on recovery medium under standard conditions

To assess the regenerative potential of the conserved rhizome microcuttings, rhizome explants were aseptically transferred to a fresh recovery medium for proliferation after 6 and 12 months of conservation. The recovery medium consisted of a full-strength MS supplemented with 1.5 mg L^−1^ BA and was incubated under standard conditions. After four weeks, the regeneration percentage as well as the number and length (cm) of shoots per explant were measured to further elucidate the influence of the conservation treatments on the overall growth and development of *P. hexandrum*. The experimental setup was replicated, and statistical analyses were employed to validate the observed trends and variations.

### Statistical analysis

The experiment was conducted in a completely randomized design (CRD) with three replicates and ten microcuttings per replicate. The data were subjected to analysis of variance (ANOVA) and the mean was compared by Duncan’s multiple range test (DMRT) at 5% level of significance using SPSS software version 20.0. The graphs were plotted using Sigma Plot 12.0 software.

## Results

### In vitro germplasm conservation of callus under slow growth conditions

**Fig. 2:**
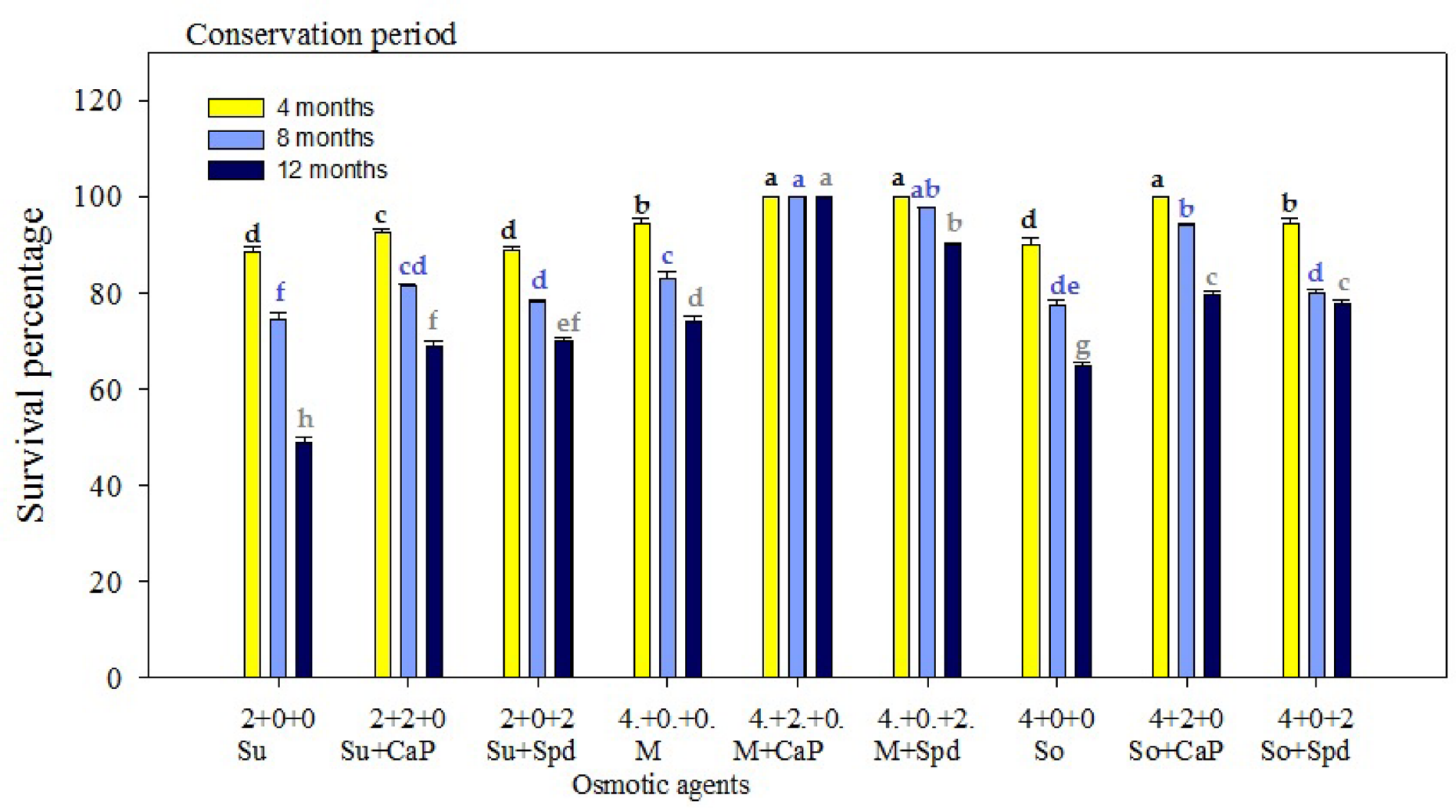
Effect of selected osmotic agents (Su: sucrose [%]; M: mannitol [%]; So: sorbitol [%]) and their combinations with calcium pantothenate (CaP [mg L^−1^]) and spermidine (Spd [mg L^−1^]) on callus survival of *P. hexandrum* in conservation medium during 4, 8, and 12 months at 5 °C. The treatments shown represent the subset of treatments selected for visualization based on their significance and relevance to the study. Means followed by similar letters are statistically at par with each other at *P* ≤ 0.05.

**Fig. 3:**
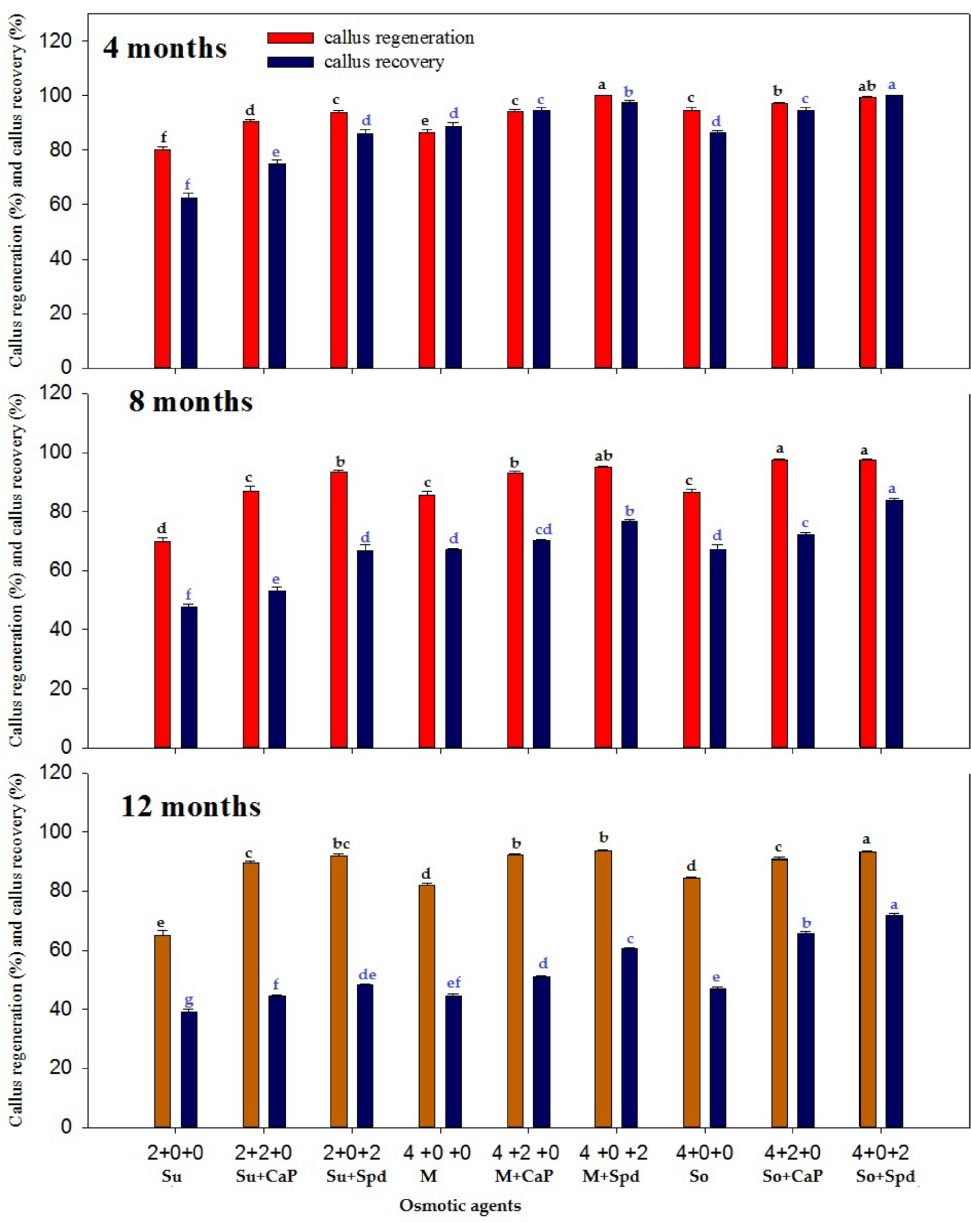
Effect of osmotic agents (Su: sucrose [%]; M: mannitol [%]; So: sorbitol [%]) and their combinations with calcium pantothenate (CaP [mgL^−1^]) and spermidine (Spd [mgL^−1^]) on callus regeneration and callus recovery of *P. hexandrum* in conservation medium during 4, 8, and 12 months at 5 ℃. Means followed by similar letters are statistically at par with each at *P* ≤ 0.05.

**Fig. 4:**
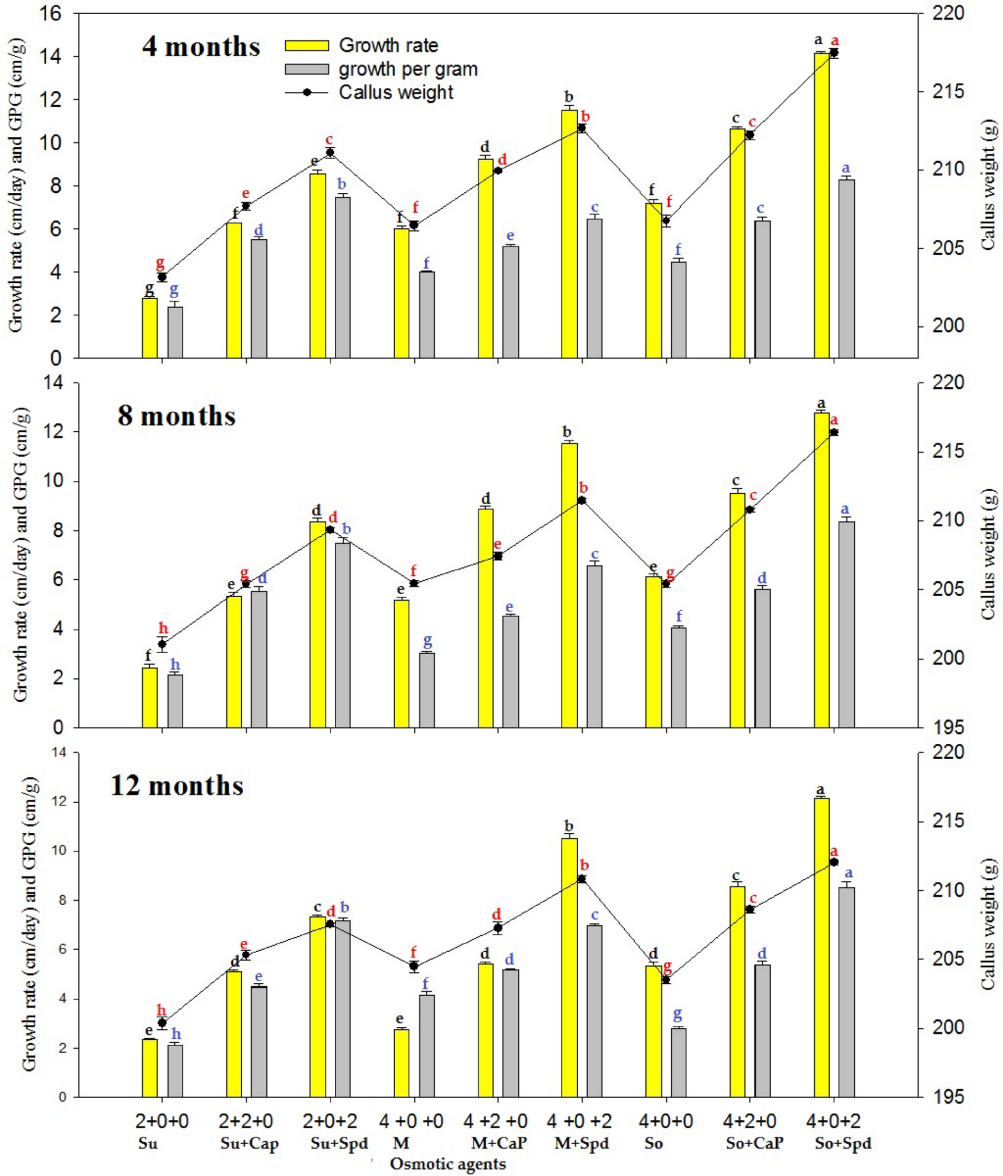
Effect of osmotic agents (Su: sucrose [%]; M: mannitol [%]; So: sorbitol [%]) and growth supplements on the callus of P. hexandrum under slow-growth conditions in conservation medium during 4, 8, and 12 months at 5 °C. Means followed by the same letters are not significantly different at *P* ≤ 0.05. CWT: Callus Weight; GRT: Growth Rate; GPG: Growth per Gram; CR_Regen%: Callus Regeneration Percentage; CR_Reco%: Callus Recovery Percentage.

**Fig. 5:**
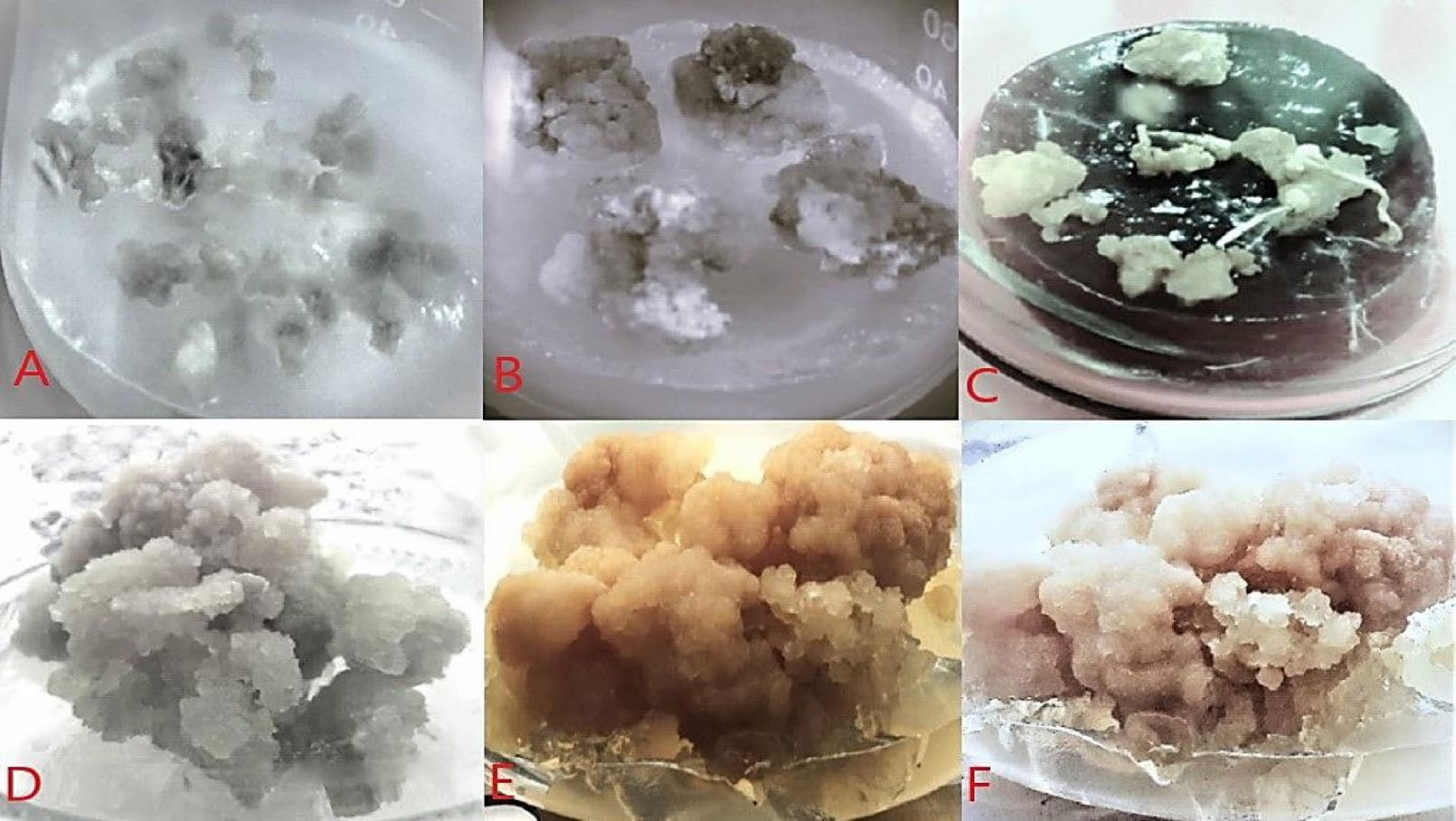
(**A**) Callus conserved for 12 months on a medium containing sucrose (2–3%), calcium pantothenate (2 mg L^−1^), spermidine (2 mg L^−1^), (**B**) Callus conserved for 12 months on a medium containing mannitol (4%), calcium pantothenate (2 mg L^−1^), spermidine (2 mg L^−1^), (**C**) Callus conserved for 12 months on a medium containing sorbitol (4%), calcium pantothenate (2 mg L^−1^), Spermidine (2 mg L^−1^), (**D**) Callus regenerated on Recovery MS medium after 12 months on sucrose treatment, (**E**) Callus regenerated on recovery MS medium after 12 months of mannitol treatment, (**F**) Callus regenerated on recovery MS medium after 12 months of sorbitol treatment.

A comparative analysis between the treatment groups and the control revealed significant improvements in survival, regeneration, and growth parameters of rhizome microcuttings and callus under slow-growth conditions. The control group, which consisted of half-strength MS medium supplemented with the standard 3% sucrose and no additional osmotic regulators or growth supplements, consistently showed lower survival percentages and reduced regeneration potential across both callus and rhizome experiments. In contrast, treatment combinations involving osmotic agents and growth regulators such as spermidine and calcium pantothenate demonstrated markedly higher performance metrics. These differences validate the functional importance of the osmotic and growth supplements tested, establishing the control as a critical baseline for evaluating treatment efficacy. The in vitro germplasm conservation experiment evaluated the effect of different supplements in the conservation medium and various conservation periods (4, 8, and 12 months) on the survival percentage, callus weight, growth rate, growth per gram, recovery, and regeneration of *P. hexandrum* callus at 5 °C (Figs. 2, 3 and 4). The results indicated that the survival percentages, callus weight, growth rate, growth per gram, recovery, and regeneration of the calli varied based on the type and concentration of substances in the conservation medium. After 4 months of conservation, the treatment with 4% sorbitol and 2 mg L^−1^ spermidine yielded the highest observed values for survival rate (100%), callus weight (217.46 ± 0.31 g), growth rate (14.13 ± 0.11 mm/day), growth per gram (8.28 ± 0.17 mm/g), callus recovery rate (99.44 ± 0.29%), and regeneration rate (100.00 ± 0.0%)(Figs. 2 and 5).

The treatment with 4% sorbitol + 2 mg L^−1^ CaP, showed a survival rate of 100 ± 0.0%, callus weight of 212.23 ± 0.29 g, growth rate of 10.63 ± 0.10 mm/day, growth/g of 6.37 ± 0.15 mm/g, callus recovery of 97.00 ± 0.57%, and regeneration percentage of 94.67 ± 0.88% (Fig. 5-F). Conversely, the lowest values for growth parameters were observed when 4% sorbitol was used without any supplement. Similarly, there was a gradual decrease in the values of growth parameters observed at 8 and 12 months of the conservation periods. The combination of 4% mannitol + 2 mg L^−1^ spermidine yielded the second-highest values for survival rate (100 ± 0.00%), callus weight (212.65 ± 0.27 g), growth rate (11.49 ± 0.25 mm/day), growth/g (6.47 ± 0.20 mm/g), callus recovery (100 ± 0.00%), and regeneration percentage (97.33 ± 0.88%) after 4 months of conservation (Figs. 3). The combination of 4% sucrose + 2 mg L^−1^ spermidine yielded the lowest values for survival rate (100 ± 0.00%), callus weight (211.10 ± 0.32 g), growth rate (8.55 ± 0.16 mm/day), growth per gram (7.44 ± 0.21 mm/g), callus recovery (93.78 ± 0.90%), and regeneration percentage (93.78 ± 0.9091%) after 4 months of conservation compared to sorbitol, mannitol, and their combinations (Figs. 2, 3 and 4).

The results showed that the best treatment for maintaining the callus quality and regeneration potential was 4% sorbitol + 2 mg L^−1^ spermidine (Figs. 4). Treatment with 4% sorbitol + 2 mg L^−1^ CaP also showed favorable results, though slightly lower than the aforementioned treatment. The lowest values for growth parameters were observed when 4% sorbitol without any supplement was used. The combination of 4% Mannitol + 2 mg L^−1^ spermidine yielded the second-highest values after 4 months of conservation (Figs. 5E).The combination of 4% sucrose + 2 mg L^−1^ spermidine yielded the lowest values after 4 months of conservation compared to sorbitol and mannitol combinations. The growth parameters exhibited a gradual decline with longer conservation periods extending to 8 and 12 months, therefore unveiling a subtle diminution in the vitality and vigor of the *P. hexandrum* callus over time (Figs. 2, 3 and 4).

### Principal component analysis

**Fig. 6:**
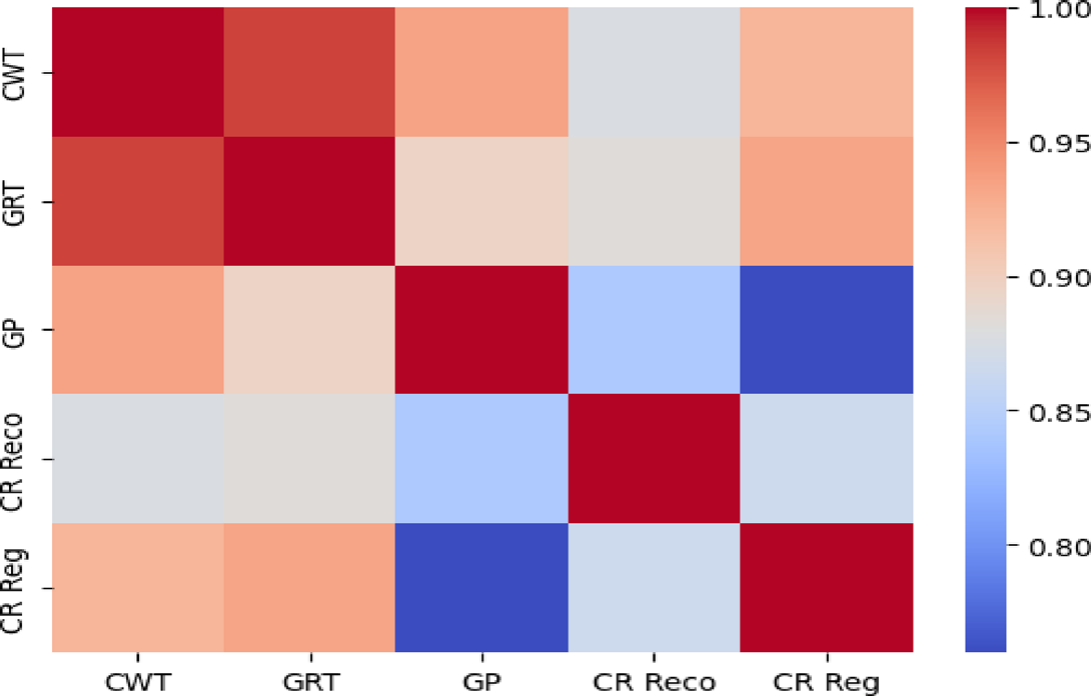
Heatmap showing the correlation between different studied traits. The sidebar represents the range of Pearson correlation coefficients ranging from − 1 to 1.

A heatmap was constructed to show the outcomes of a correlation analysis between callus regeneration percentage (CR Reg %), callus recovery percentage (CR Reco %), callus growth per gram (GPG), growth rate (GRT), and callus weight (CWT) of the germplasm. A positive correlation was observed between CWT and GRT, GP, and CR Reg. Conversely, CR Reco revealed a negative correlation with all the measured traits (Fig. 6).

**Fig. 7:**
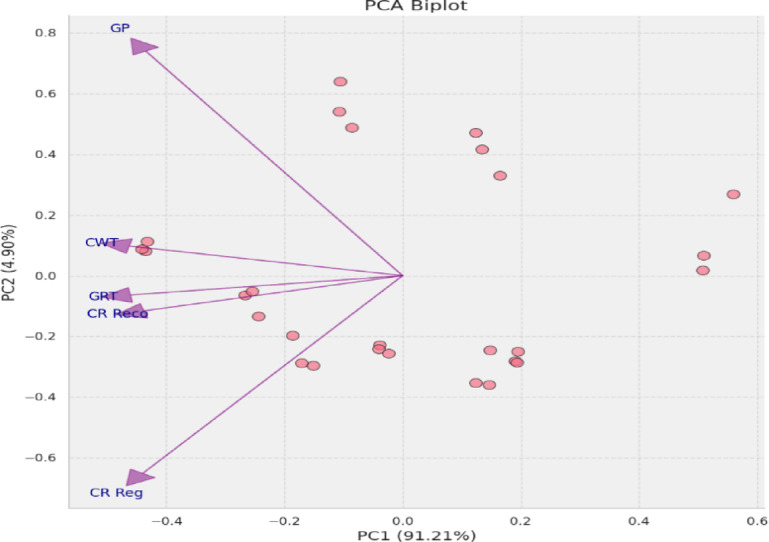
A PCA biplot encapsulating multidimensional trait data. Vectors represent the five measured traits viz. callus growth per gram (GPG), callus regeneration percentage (CR Reg%), callus recovery percentage (CR Reco%), callus growth (CRT), and callus weight (CWT), with their orientation and magnitude corresponding to their contribution and correlation to the principal components. The scatter of data points reflects the distribution of individual samples across the derived trait space.

The PCA biplot analysis of the studied traits revealed that the first principal component (PC1) accounted for the majority of the variance (91.21%) with an eigenvalue of 4.73, and the second principal component (PC2) contributing with 4.90%, and an eigenvalues of 0.25, indicating a predominant dimension of variation among the samples, primarily related to traits associated with callus weight (CWT), callus rate (GRT), callus recovery percentage (CR Reco), and callus growth per gram (Fig. 7).

### In vitro germplasm conservation of rhizome explants of *P. hexandrum*

**Fig. 8:**
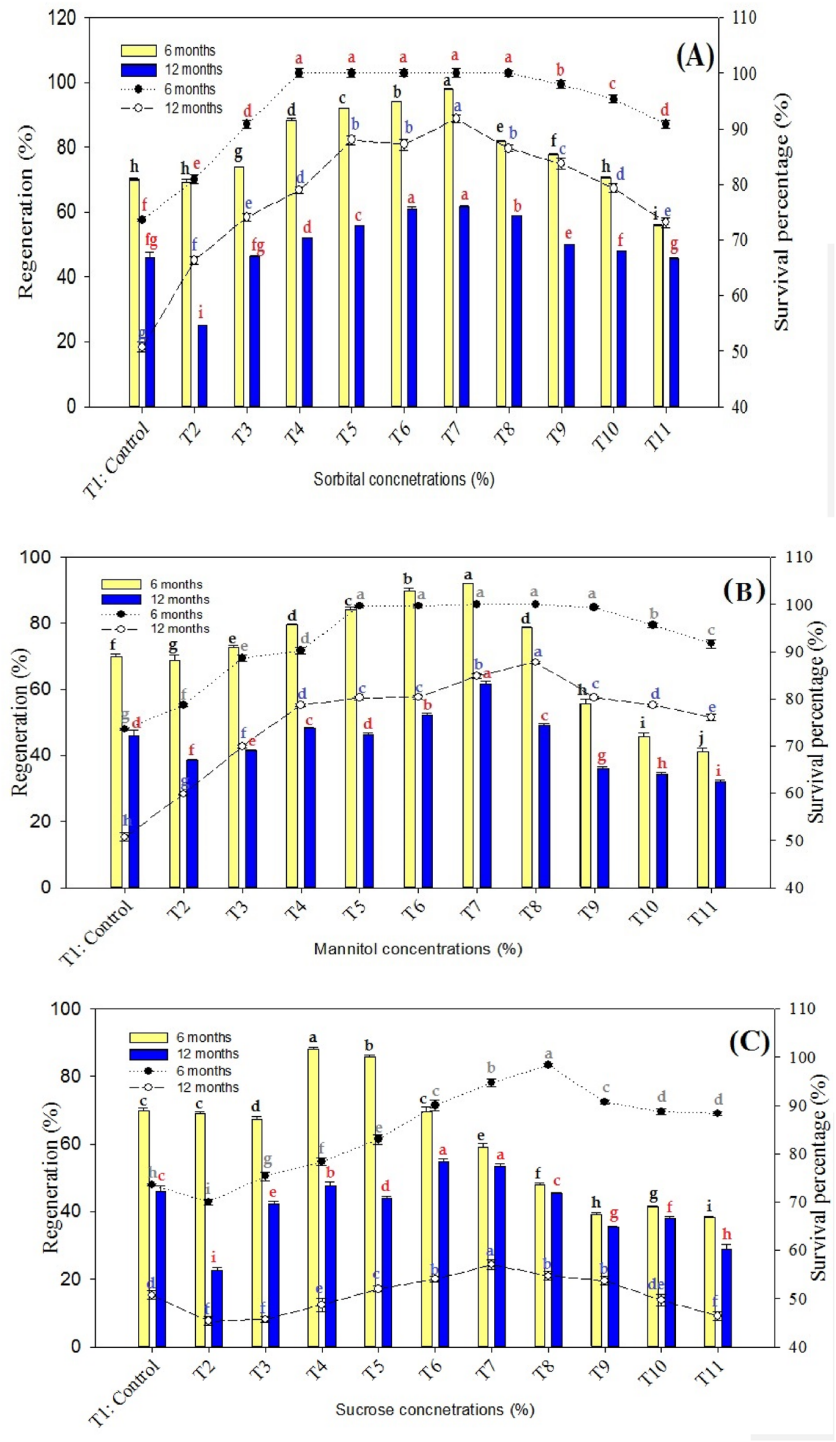
(**a**) Effect of different concentrations of sorbitol in combination with spermidine (1.5 mg L^−1^), calcium pantothenate (1.5 mg L^−1^), and BA (1.5 mg L^−1^) on the in vitro recovery and survival of *P. hexandrum* rhizome explants conserved at 5 °C in the dark for 6 and 12 months. (**b**) Effect of different concentrations of mannitol in combination with spermidine (1.5 mg L^−1^), calcium pantothenate (1.5 mg L^−1^), and BA (1.5 mg L^−1^) on the in vitro recovery and survival of *P. hexandrum* rhizome explants conserved at 5 °C in the dark for 6 and 12 months. (**c**) Effect of different concentrations of sucrose in combination with spermidine (1.5 mg L^−1^), calcium pantothenate (1.5 mg L^−1^), and BA (1.5 mg L^−1^) on the in vitro recovery and survival of *P. hexandrum* rhizome explants conserved at 5 °C in the dark for 6 and 12 months.

**Fig. 9:**
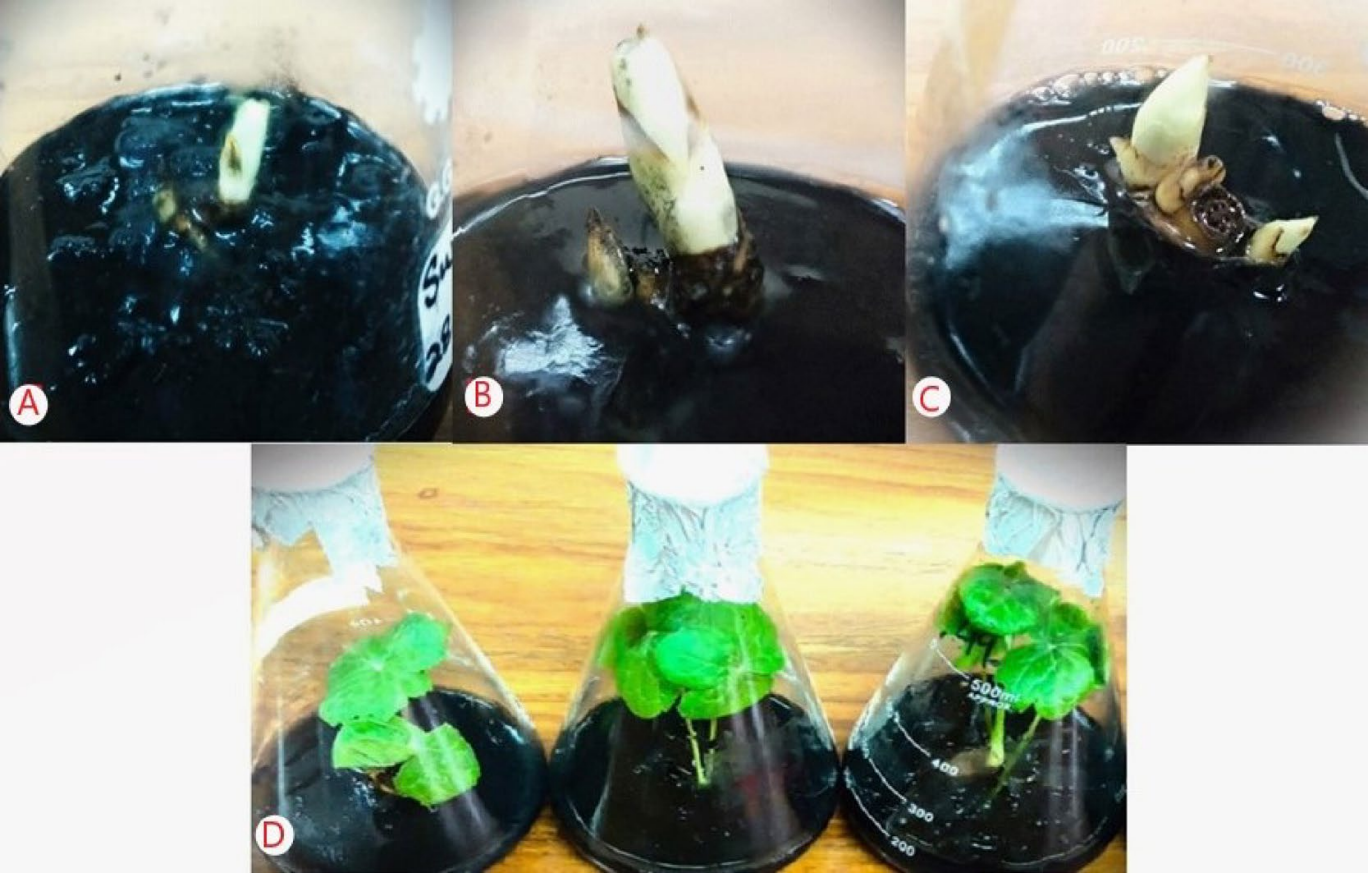
(**A**) Rhizome explants cultured on a medium containing sucrose (2.5–10.5 mg L^−1^), calcium pantothenate (2 mg L^−1^), spermidine (2 mg L^−1^), activated charcoal (3 g L^−1^), (**B**) Rhizome explants cultured on a medium containing mannitol (2.5–10.5 mg L^−1^), calcium pantothenate (2 mg L^−1^), spermidine (2 mg L^−1^), AC (3 g L^−1^), (**C**) Rhizome explants cultured on a medium containing sorbitol (2.5–10.5 mg L^−1^), calcium pantothenate (1.5 mg L^−1^), spermidine (1.5 mg L^−1^), AC (3 g L^−1^), (**D**) Rhizome explants regeneration on recovery MS medium after 12 months conservation supplemented with BA (1.5 mg L^−1^), AC (3 g L^−1^).

 The current study evaluated the effects of various osmotic regulators and growth supplements on the survival, regeneration percentage, shoot number, and shoot length of *P. hexandrum* rhizome explants conserved at 5 °C in the dark for 6 and 12 months (Figs. 8 and 9). Significant variation was observed among the treatments, with sorbitol-based media showing the most promising results. Specifically, sorbitol at 5.5%, in combination with spermidine (2 mg L^−1^), calcium pantothenate (3 mg L^−1^), and BA (1.5 mg L^−1^), resulted in the highest values for shoot length (5.7 ± 0.05 cm), shoot number (4.87 ± 0.04), regeneration percentage (97.86 ± 0.26%), and survival percentage (100.00 ± 0.00%) after 6 months. Even after 12 months of storage, this treatment maintained the highest survival (91.77 ± 0.73%) and regeneration values (61.60 ± 0.23%), indicating its suitability for long-term conservation.

Sorbitol showed a positive correlation with regeneration percentage, shoot number, and shoot length, since the values increased with increasing sorbitol concentration up to 5.5%. The highest values for shoot length (5.7 ± 0.051 cm), shoot number (4.87 ± 0.043), regeneration percentage (97.86 ± 0.261%), and survival percentage (100.00 ± 0.00%) at 6 months were obtained with sorbitol (5.5%) combined with spermidine (2 mg L^−1^), calcium pantothenate (3 mg L^−1^), and BA (1.5 mg L^−1^). This treatment also resulted in the highest values for shoot length (4.21 ± 0.082 cm), shoot number (3.53 ± 0.008), regeneration percentage (61.60 ± 0.23%), and survival percentage (91.77 ± 0.73%) after 12 months of conservation, with a mean survival percentage of 95.89 ± 0.36% (Figs. 8-A and 9-C). This suggests that sorbitol has superior osmoprotectant properties and can enhance the survival of rhizome explants.

Spermidine and calcium pantothenate improved the survival percentage of rhizome explants when added to any of the aforementioned sugars, with spermidine showing a more consistent effect than calcium pantothenate. The survival percentage of rhizome explants decreased as the conservation period increased from 6 to 12 months, except for mannitol (2%)/spermidine (2 mg L^−1^)/calcium pantothenate (3 mg L^−1^)/BA (1.5 mg L^−1^) combination, which maintained maximum regeneration and survival percentage throughout the conservation period (Figs. 8-B and 9-B). This demonstrates that certain combinations of spermidine and calcium pantothenate have a significant impact on the survival of rhizome explants.

The results showed that the survival percentage of conserved rhizome explants of *P. hexandrum* was significantly influenced by the addition of calcium pantothenate, spermidine, and by the type of added sugar (sucrose, sorbitol, and mannitol) to the conservation medium at 5 °C under complete darkness. The results also show that the conservation period (6 or 12 months) affects the survival percentage of conserved rhizome explants of *P. hexandrum* and that sorbitol and mannitol are better osmoprotectants than sucrose for enhancing rhizome explant survival. It was observed that spermidine and calcium pantothenate can improve the survival percentage of rhizome explants when combined with any of the sugars, therefore providing insights into the optimal conditions for the conservation of *P. hexandrum* rhizome explants.

Mannitol and sorbitol supplementation consistently demonstrated significant growth enhancement and improved survival percentages at 6 and 12 months, with sorbitol exhibiting the most pronounced effects being the most effective osmotic agent for enhancing the explant recovery, followed by mannitol and sucrose. Sorbitol and mannitol significantly improved the regeneration percentage, shoot number, and shoot length compared to sucrose. The optimal concentration for all osmotic agents was 5.5%, which resulted in the highest mean values for all parameters.

## Discussion

This study investigated the efficacy of osmotic agents and growth supplements in enhancing the long-term in vitro conservation of *P.hexandrum* calli and rhizome explants at 5 °C. Among the osmotic agents tested, Sorbitol proved to be the most effective osmotic agent, with 4–5.5% combined with 2 mg L^−1^ spermidine and 3 mg L^−1^ calcium pantothenate significantly improving survival and regeneration in both calli and rhizome explants. Sorbitol acts as a non-metabolizable sugar alcohol that helps maintain osmotic balance without triggering metabolic stress. Its ability to stabilize cellular membranes and prevent oxidative damage under cold stress conditions makes it highly suitable for long-term tissue conservation. Sorbitol and mannitol were identified as effective osmotic agents in our study based on their consistent and superior phenotypic performance specifically, their ability to significantly enhance survival rates, regeneration percentages, and growth parameters of *P. hexandrum* callus and rhizome explants during slow-growth storage^46^. Sucrose had negative effects on increasing concentration, likely due to rapid metabolism and ROS buildup^47,48^. The callus weight and growth rate remained stable over time, while regeneration of callus was declined by 36%. This paradox likely reflects cold-induced metabolic prioritization, where basic cellular maintenance and biomass accumulation are preserved, but energy-intensive processes like organogenesis are suppressed. Similar findings in *Panax ginseng* cultures showed that cold storage maintained callus integrity but delayed regeneration. Likewise, *P. hexandrum* required post-storage supplementation with growth regulators to restore regeneration potential^38,44^.

The contrasting effects of sucrose and mannitol are concentration and context-dependent. At high concentrations or during prolonged cold storage, both sugars may act as growth inhibitors due to osmotic stress. However, mannitol at moderate levels (4–5.5%) functioned as a non-metabolizable osmoprotectant, enhancing survival and regeneration by maintaining osmotic balance without inducing metabolic strain. In contrast, sucrose, even at lower concentrations (2–3%), showed negative effects likely due to rapid metabolism, medium acidification, and ROS accumulation. Thus, the physiological outcome depends on sugar type, concentration, exposure duration, and tissue state^45,47–50^. A negative correlation between regeneration and other growth traits also supports the hypothesis of a physiological trade-off under stress conditions^51^. In rhizome explants, the best regeneration outcomes were obtained using the sorbitol-spermidine-CaP-BA combination. Although mannitol also improved survival, its effects were slightly less than those of sorbitol^52,53^. These results emphasize the need to customize the conservation medium based on explant type, duration of storage, and intended recovery potential.

TTC assay results confirmed that most calli remained metabolically viable (red-stained), despite reduced regeneration after prolonged storage. This highlights that viability does not guarantee morphogenic competence. Factors such as senescence, epigenetic changes, or metabolic byproduct accumulation may suppress regeneration while allowing biomass growth^19,36,38,54^. The observed negative correlation between CR Reco% and other traits (Fig. 6) suggests a physiological trade-off, with resources shifting toward maintenance rather than organogenesis^36,38,51,55,56^. These findings reinforce the concept that long-term cold storage sustains metabolic integrity but can compromise totipotency and regenerative performance of conserved tissues unless appropriately rejuvenated during recovery^56,57^.

Similarly, adding 2 mg L^−1^ spermidine and CaP consistently improved rhizome viability, with spermidine achieving 91.3% survival after 12 weeks. The combination of CaP and 4% sorbitol further increased survival to 95.8%, demonstrating superior efficacy for long-term tissue conservation^57–60^. Contrarily, using sucrose (2%) alone in the conservation medium resulted in a decline to 50% mean survival after 12 weeks, indicating inadequate protection against prolonged conservation stress^55^. Sucrose hydrolysis can lead to glycolytic overflow, resulting in ATP depletion in conserved tissues^27^. Moreover, sucrose has been documented to induce reactive oxygen species (ROS) accumulation such as hydrogen peroxide (H₂O₂) and malondialdehyde (MDA) in slow-growth cultures. Media acidification caused by sucrose (pH ≤ 4.5) has also been shown to impair nutrient uptake during storage. In contrast, sugar alcohols like sorbitol and mannitol help maintain osmotic balance without interfering with primary metabolism, making them more suitable for long-term conservation^28,55,56^. Sorbitol, in particular, has demonstrated significant potential due to its ability to induce osmotic stress responses, which enhances cellular resilience and longevity. Studies have shown that sorbitol activates stress-response pathways, suggesting its protective role in plant cells under in vitro conservation. Additionally, its ability to modulate oxidative stress supports its utility in preserving plant tissues by mitigating reactive oxygen species (ROS) accumulation^61^. Furthermore, in vitro biotechnology offers sustainable approaches for conserving plant genetic resources, reinforcing the importance of slow-growth strategies^62^. Research on slow-growth storage techniques has proven effective for various species, including *P. alba* and *H. speciosa*^63,64^, where osmotic regulators-maintained viability over extended periods. Thus, incorporating sorbitol in conservation protocols provides a promising method for preserving endangered plant germplasm while minimizing genetic and physiological deterioration.

By ensuring the long-term availability of genetically stable material for research purposes, this work aligns with global targets such as the Global Strategy for Plant Conservation (GSPC) Target 8 and supports biotechnological interventions in medicinal plant conservation beyond the regional scope of Pakistan. Despite the success of the slow-growth protocol, two key limitations remain. First, the absence of a low-temperature control group without osmoprotectants limits the ability to isolate the specific effects of the added supplements. Second, using fixed concentrations of spermidine and calcium pantothenate across treatments prevents assessment of their individual contributions. Future research should incorporate variable levels of these regulators to better understand their roles. In conclusion, this study demonstrates that sorbitol and mannitol are effective osmoprotectants for the long-term in vitro conservation of *P. hexandrum*. Their combination with spermidine and calcium pantothenate significantly enhanced tissue viability, metabolic integrity, and post-storage regeneration. These findings offer practical insights for improving tissue culture-based conservation protocols for endangered medicinal plants.

## Conclusions

*P. hexandrum* Royle is a valuable medicinal plant facing extinction in the Himalayan regions of Pakistan. The slow growth storage (SGS) technique successfully demonstrated its effectiveness in preserving *P. hexandrum* germplasm for short and medium-term periods without subculturing. Our findings highlight the efficacy of sorbitol (5.5%), spermidine (2 mg L^−1^), calcium pantothenate (3 mg L^−1^), and BA (1.5 mg L^−1^) in maintaining viability for both callus and rhizome explants. This study developed an effective in vitro conservation protocol for *P.hexandrum*, an endangered medicinal plant, using slow growth storage techniques. The combination of sorbitol, mannitol, spermidine (2 mg L^−1^), and calcium pantothenate (2 mg L^−1^) significantly enhanced tissue viability, stress tolerance, and long-term survival of both callus and rhizome explants. The optimized medium, using 4% sorbitol or mannitol, proved crucial for maintaining genetic stability and cell viability during extended cold storage. This study provides a sustainable method for conserving the endangered medicinal plant i.e. *P. hexandrum germplasm*, ensuring the availability of genetically stable plant material for future research and pharmaceutical applications. However, limitations such as the lack of long-term viability data and the need for field trials should be addressed in future studies. We recommend further research to explore the effects of various environmental conditions on the growth and regeneration of *P. hexandrum*, as well as the potential for commercial applications of its bioactive compounds. However, the study did not independently assess the effects of spermidine and calcium pantothenate, which limits our ability to fully disentangle their individual versus combined contributions. Future research should adopt a factorial design to evaluate the specific roles and interactions of each additive in enhancing explant survival. Future studies should incorporate direct mechanistic evidence such as HPLC-based osmolyte profiling and RNA-seq analysis of stress-responsive genes (e.g., *P5CS*, *LEA*) to better understand the physiological and molecular effects of sorbitol, mannitol, and sucrose under slow growth storage conditions.

## Data Availability

Data is provided within the manuscript.
